# Construction Notes

**Published:** 1897-01-02

**Authors:** 


### CONSTRUCTION NOTES.

**New Infectious Diseases Hospital at Kirkcaldy.**

The ground surrounding this new building embraces an area of four acres. The buildings are of brick, with hollow walls, and all modern features, recognised as the best in a first-class hospital, will be embraced in this work. Accommodation is provided for twenty beds for adults, with day-rooms sufficient for three additional beds on emergency. The cost of the buildings, with all fittings, will be about £7,500. Messrs. Campbell, Douglas, and Morrison are the architects.

